# Ringing phenomenon in chaotic microcavity for high-speed ultra-sensitive sensing

**DOI:** 10.1038/srep38922

**Published:** 2016-12-14

**Authors:** Lei Chen, Qian Liu, Wei-Gang Zhang, Keng C. Chou

**Affiliations:** 1 Key laboratory of Optical Information Science and Technology, Ministry of Education, Institute of Modern Optics, Nankai University, Tianjin 300071, China; 2 Department of Chemistry, University of British Columbia, Vancouver, British Columbia V6T 1Z1, Canada

## Abstract

The ringing phenomenon in whispering-gallery-mode (WGM) microcavities has demonstrated its great potential for highly-sensitive and high-speed sensing. However, traditional symmetric WGM microcavities have suffered from an extremely low coupling efficiency via free-space coupling because the emission of symmetric WGMs is non-directional. Here we report a new approach for high-speed ultra-sensitive sensing using the ringing phenomenon in a chaotic regime. By breaking the rotational symmetry of a WGM microcavity and introducing chaotic behaviors, we show that the ringing phenomenon in chaotic WGM microcavities extends over both the positive and the negative frequency detune, allowing the ringing phenomenon to interact with analytes over a much broader bandwidth with a reduced dead time. Because the coupling of the chaotic microcavity is directional, it produces a significantly higher signal output, which improves its sensitivity without the need of a fiber coupler.

Whispering-gallery-modes (WGMs) are specific modes (or resonances) of a wave that are confined inside a concave cavity because of total internal reflection^1^. Many unique properties of optical WGMs, e.g. ultra-high quality (Q) factors and small cavity sizes, make them suitable for a variety of applications, such as all-optical switches^1^, laser sources^2^,^3^, filters^4^,^5^, sensors^4^,^6^,^7^, and quantum computing^8^. WGM-based highly-sensitive sensing has drawn great attention because WGM cavities are able to confine the optical field in a very small space for an extended time period, which greatly enhances the interaction between the optical field and the analyte^4^,^6^,^9^,^10^. For highly-sensitive sensing, a frequency (wavelength)-variable laser sweeps the output frequency over the resonance of a WGM microcavity, and the resonant frequency is measured^11^. In the presence of electromagnetic fields^4^, nanoparticles^6^, or molecules^9^,^12^, which interact with the evanescent field of the microcavity, the resonant frequency may shift, broaden, or split^6^,^9^. Because of the ultra-high Q factor of WGMs, the detection is highly sensitive.

When the frequency chirp rate of the laser is increased for high-speed sensing, a ringing phenomenon (RP) in the mapped WGM spectrum occurs^3^. Traditionally, the RP in a symmetric WGM microcavity is used to measure the Q-factor and the mode-coupling strength^4^,^13^. Recently it was reported that sensing based on the RP could achieve a very high speed and was not sensitive to the wavelength drift of the laser^14^. However, traditional symmetric WGM microcavities have suffered from their extremely low coupling and collection efficiencies because the emission of symmetric WGM microcavities is non-directional^15^. To enhance the coupling and collection efficiencies, several evanescent wave couplers, such as tapered fibers^11^, prisms^16^, or waveguides^17^, have been employed but the size and the special requirements of these auxiliary couplers have greatly hindered their applications^10^,^18^.

The RP in chaotic WGM microcavities reported in this study provides a feasible approach to overcome the coupling and collection deficiencies for high-speed ultra-sensitive sensing. It has been reported that the emission of a WGM microcavity becomes directional if the rotational symmetry of the microcavity is broken^19^,^20^. However, breaking the symmetry introduces chaotic behaviors into the microcavity^21^,^22^, and a detailed study on the RP in a chaotic WGM microcavity has not yet been reported.

In this study, we theoretically investigate the RP in a chaotic WGM microcavity and its advantages for high-speed ultra-sensitive sensing over a symmetric WGM microcavity. The chaotic behaviors of the microcavity were introduced by breaking the rotational symmetry of the WGM microcavity^20^. Beside the advantage of directional output and high coupling efficiency for a higher sensitivity, we found that the RP spectra of the chaotic WGM microcavity had a much broader spectral bandwidth, which significantly reduced the dead time for high-speed sensing. The RP in the chaotic WGM microcavity extends over both the positive and the negative frequency detuning while the RP in a symmetric WGM microcavity occurs only at the positive detune frequency^2^,^11^,^23^,^24^.

## Results

### Theoretical Background

**Figure 1:**
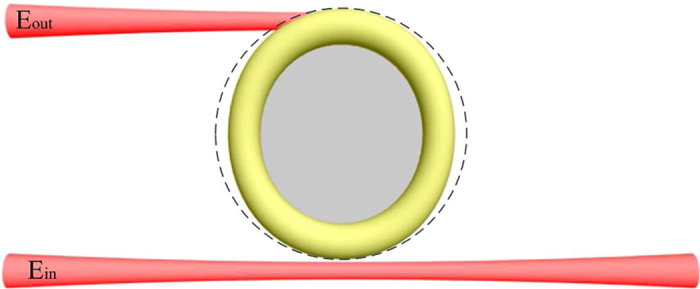
Schematic illustration of the deformed chaotic microcavity (yellow). The dashed line indicates a perfect circle. The red beams are the input (*E*_*in*_) and output beams (*E*_*out*_).

The chaotic microcavity investigated in the current study is a deformed WGM microcavity as shown in Fig. 1. The geometry of the cavity is described as follows^21^


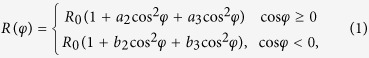


with *R*_0_ = 45 *μ*m, *a*_2_ = −0.1329, *a*_3_ = 0.0948, *b*_2_ = −0.0642, and *b*_3_ = −0.0224.

Previous studies on the RP of symmetric WGM microcavities were described by a simple harmonic oscillator model established by Haus^2^,^6^,^25^. However, this approach is only suitable for a linear periodic system, such as a WGM microcavity with a rotational symmetry. Chaotic systems are known for their non-periodic and nonlinear characteristics^26^. Therefore, the simple harmonic oscillator model is not valid for a chaotic system.

For a more general discussion, a WGM microcavity can be regarded as a frequency filter described by a transfer function *h*(*t*), which relates the input electric field *x*(*t*) and the output electric field *y*(*t*) as follows.


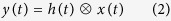


where ⊗ denotes a convolution in the time domain. By using the convolution theorem, it can be shown that


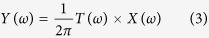


where *Y*(*ω*), *T*(*ω*), and *X*(*ω*) are the Fourier transformations of *y*(*t*), *h*(*t*), and *x*(*t*), respectively. The output intensity in the time domain is


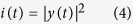


which is the inverse Fourier transformation of


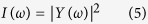


For a linear system, *h*(*t*) and *T*(*ω*) are independent of the input^27^, but *h*(*t*) and *T*(*ω*) in the current chaotic system depend on the amplitude and the phase of the input field. When an electric field with a linear chirp is coupled to the chaotic WGM microcavity shown in Fig. 1 21,22, the system can be described by the stationary Schrodinger equation with 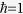 22


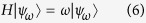


where *H* is the Hamiltonian of the chaotic microcavity, and 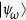 are the eigenstates. 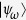 can be written in two components


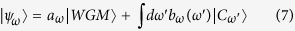


where 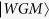 are the eigenstates of a WGM microcavity with a rotational symmetry, 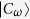 are the chaotic WGM modes in the asymmetric microcavity, and 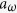 and 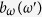 are the coefficients of the symmetric and the chaotic WGM modes, respectively. The chaotic WGM modes 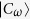 are continuous within the input bandwidth because the chaotic WGM modes originate from the irregular reflection of the asymmetric cavity^22^,^28^. In this system, the chaotic WGM modes may couple to the conventional WGM modes via dynamic tunneling^28^. Therefore the Hamiltonian satisfies^21^,^22^,^29^


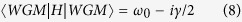



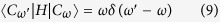



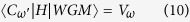


where *ω*_0_ is the WGM resonant frequency, *γ* is the decay rate describing the intrinsic loss and the chaotically-assisted tunneling loss, *Vω* describes the coupling between 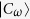 and 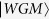. It can be shown that the coefficient in Eq. (7) can be written as 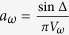 and 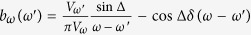 with 
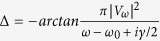
29. For simplicity, one can define 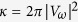, 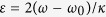, and 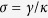, then 
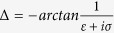
. The transmission function can be expressed as^21^,^29^


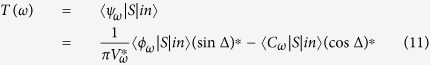


where *S* is the transmission operator; 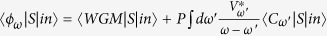. Eq. (11) can be further simplified if we define 
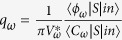
. Then


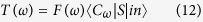


with^21^


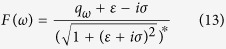



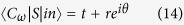



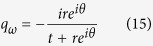


where *r* and *θ* are the amplitude factor and the phase difference of the forward-emitted fields between the chaotic and symmetric WGM microscavities, respectively. The values of *r* and *θ* are determined by the experimental geometry.

## Discussion

**Figure 2:**
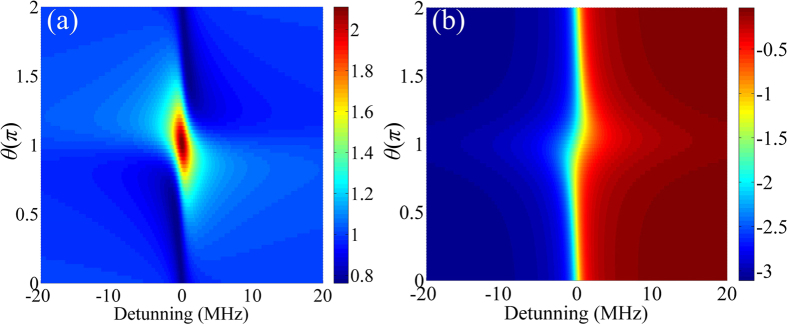
Amplitude (**a**) and phase (**b**) profiles of *F*(*ω*) in Eq. (13).

To numerically simulate the RP in a chaotic WGM microcavity, we let 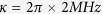, 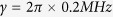, and *r*/*t* = 2 which are within typical experimental values. Since the term 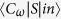 in Eq. (14) is a complex number without any particular features, for simplicity, our discussions focus on *F*(*ω*). Two characteristics of the chaotic WGM modes are distinguishably different from those of symmetric WGM modes. First, the coefficient *F*(*ω*) exhibits a Fano-like profile^21^,^28^, as shown in Fig. 2a, while the symmetric WGM modes are known to have Lorentz profiles^3^,^11^. Secondly, the chaotic WGM modes have a normal dispersion (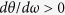), as shown in Fig. 2b, in contrast to the anomalous dispersion of symmetric WGM modes^3^,^11^.

Assume the input field has a linear frequency chirp^30^


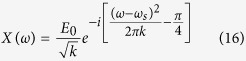


where *E*_0_ is the amplitude of the chirped laser, *ω*_*s*_ is the starting angular frequency, and *k* is the chirp rate (*df*/*dt*). By substituting Eq. (16) into Eq. (3), the mapped spectrum as a function of the frequency detuning can be obtained using the output intensity *i*(*t*) described in Eq. (4).

**Figure 3:**
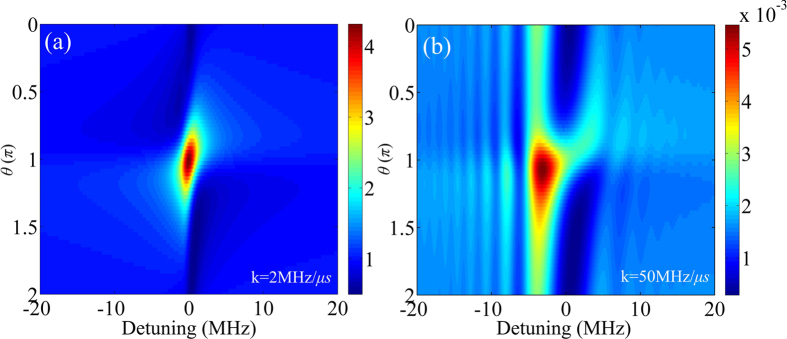
Mapped RP spectra as functions of *θ* with the chirp rate *k* = 2 MHz/*μ*s (**a**) and 50 MHz/*μ*s (**b**).

In general, the mapped spectrum of the chaotic WGM microcavity is a function of *θ* given in Eqs (14)–(15), 34, which can be adjusted by the experimental geometry. Figure 3a shows that the Fano-like profile in Fig. 2a is mostly preserved when the input field has a low chirp rate. When the chirp rate is high, the Fano-like profile disappears, and the RP appears, as shown in Fig. 3b.

**Figure 4:**
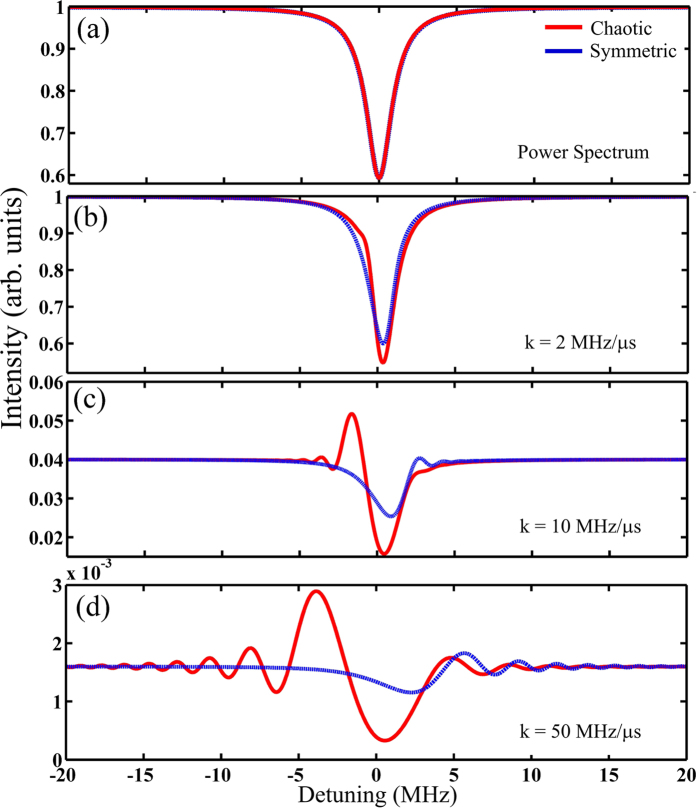
(**a**) Power spectra of the chaotic (red) and symmetric (blue) WGM microcavities. The mapped RP spectra of the chaotic (red) and the symmetric (blue) WGM microcavities with the chirp rate at 2 MHz/*μ*s (**b**), 10 MHz/*μ*s (**c**), and 50 MHz/*μ*s (**d**).

To better demonstrate the difference between the chaotic and symmetric microcavities, the mapped RP spectra were compared. Figure 4 shows the mapped spectra of the chaotic WGM microcavity (red curves) with *θ* = 0 and the symmetric WGM microcavity (blue curves). At *θ* = 0, the Fano-like profile of the chaotic WGM modes is similar to the Lorentz profile of the symmetric WGM modes^3^,^11^. The comparison was carried out with similar coupling and collection efficiencies for both microcavities so that the transmitted power spectra are similar, as shown in Fig. 4a. Since the coupling and emission of the chaotic WGM microcavity is directional, its coupling and collection efficiencies is much higher than those of a symmetric WGM microcavity. The similar transmitted power spectrum, shown in Fig. 4a, is only possible for a symmetric WGM with an evanescent wave coupler, such as tapered fibers^11^, prisms^16^ or waveguides^17^. Therefore, the following is a comparison between a chaotic WGM microscavity with free-space coupling and a symmetric WGM microcavity with a high-efficiency evanescent wave coupler.

Figure 4b shows that there is only a small difference between the chaotic and symmetric WGM outputs with a chirp rate of 2 MHz/*μ*s. Figure 4c and d show that the ringing ripples in the chaotic and symmetric WGM spectra significantly deviate from each other as the chirp rate increases. There are two advantages of the chaotic WGM microcavity for high-speed ultra-sensitive sensing. First, the RP of the chaotic WGM microcavity extends over both the positive and negative detuning, while those of the symmetric WGM microcavity only appear in the positive detuning. This allows the RP to interact with the analytes over a much broader bandwidth. The frequency detune presented in Fig. 4 is calculated with respect to the resonant frequency of the symmetric WGM mode. The broader RP spectrum of the chaotic WGM microcavity can be understood by the lower Q factor (broader bandwidth) of the chaotic microcavity. A symmetric WGM microcavity has a higher Q factor; therefore, its intensity buildup time is longer for a detuned input frequency. At a high chirp rate, the symmetric WGM microcavity has little response to a negative detune frequency. On the other hand, the chaotic WGM microcavity has a relatively quick response time for a negative detune frequency. Secondly, the chaotic microcavity produces a higher output at a higher chirp rate, which will improve the signal-to-noise ratio for high-speed sensing.

**Figure 5:**
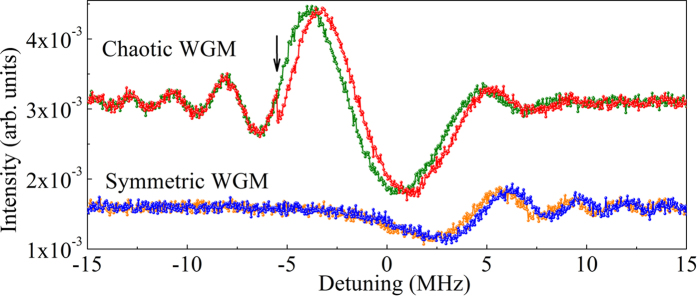
Green and orange curves show the mapped RP spectra of the chaotic and the symmetric WGM microcavities, respectively, without adsorbates. Red and blue curves show the simulated RP spectra with a molecule adsorbed on the microcavities when the frequency detune equals to −5 MHz, as indicated by the black arrow.

A numerical simulation was carried out to demonstrate the improved sensitivity and time resolution of the chaotic WGM modes. It has been previously shown that the adsorption of an interleukin-2 protein on a symmetric microcavity shifted the WGM resonant wavelength by roughly 0.01 picometer^31^. Figure 5 shows the simulated RP spectra changes with an adsorbate-induced resonant wavelength shift of 0.01 picometer for both the chaotic and symmetric WGM modes. In this simulation, a laser frequency chirp rate of 50 MHz/*μ*s was used, and a Gaussian white noise of −20 dB was added. The green and orange curves represent a single scan without any adsorbate for the chaotic and symmetric WGM microcavities, respectively. The red and blue curves are the spectra for the chaotic and symmetric WGM microcavities, respectively, with a single molecule adsorbed at −5 MHz, as indicated by the arrow in Fig. 5. The frequency shift of the RP spectrum induced by the adsorbate is clearly visible in the chaotic RP spectrum (red curve). However, the event happens in the dead zone of the symmetric WGM, and the frequency shift is visible later only in the positive frequency detune. With a frequency chirp rate of 50 MHz/*μ*s, this corresponds to a 0.2 *μ*s delay. Because of the larger amplitude of the chaotic WGM mode, the frequency-shifted spectrum is well-separated from the original spectrum. In contrast, the spectral difference in the symmetric WGM spectrum is much smaller.

In summary, we investigated the RP in chaotic WGM microcavities by treating it as a passive optical filter. The RP spectra in the chaotic and the symmetric microcavities significantly deviate from each other as the chirp rate increases. While the RP in the symmetric WGM microcavities appears only in the positive frequency detune, the RP of the chaotic WGM microcavity extends over both the positive and the negative frequency detune, which reduces the dead time for high-speed sensing. The chaotic WGM microcavity also produces a significantly higher RP output, which increases the signal-to-noise ratio for ultra-sensitive sensing without the need of a fiber coupler.

## Methods

The numerical simulation was carried out by using Matlab 2012a. 1,000,001 data points were used in the inverse Fourier transformation.

## Additional Information

**How to cite this article**: Chen, L. *et al*. Ringing phenomenon in chaotic microcavity for high-speed ultra-sensitive sensing. *Sci. Rep.* **6**, 38922; doi: 10.1038/srep38922 (2016).

**Publisher's note:** Springer Nature remains neutral with regard to jurisdictional claims in published maps and institutional affiliations.
